# Supplementary material for: Complex‐centric proteome profiling by SEC‐SWATH‐MS
Source: Mol Syst Biol. 2019 Jan 14;15(1):e8438. doi: 10.15252/msb.20188438 (PMC6346213; doi:10.15252/msb.20188438)
Supplement: Supplementary file 8 — Dataset EV7 [file MSB-15-e8438-s008.zip › feature_plots_string/Q5T200.pdf]

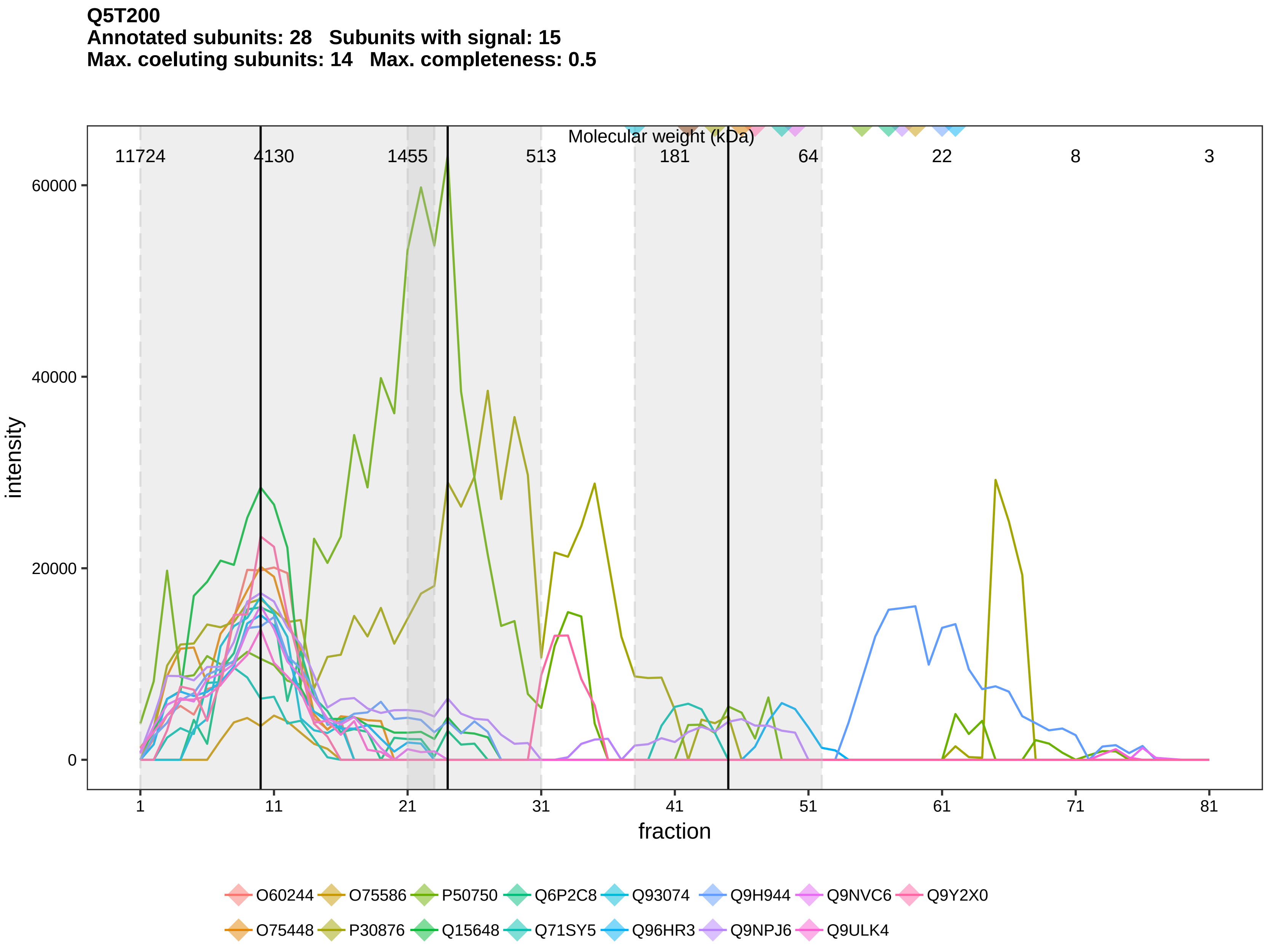
Q5T200
Annotated subunits: 28 Subunits with signal: 15
Max. coeluting subunits: 14 Max. completeness: 0.5
Molecular weight (kDa)
11724
4130
1455
513
181
64
22
8
3
60000
40000
20000
0
intensity
1
11
21
31
41
51
61
71
81
fraction
O60244
O75586
P50750
Q6P2C8
Q93074
Q9H944
Q9NVC6
Q9Y2X0
O75448
P30876
Q15648
Q71SY5
Q96HR3
Q9NPJ6
Q9ULK4
